# The prehistory of biology preprints: A forgotten experiment from the 1960s

**DOI:** 10.1371/journal.pbio.2003995

**Published:** 2017-11-16

**Authors:** Matthew Cobb

**Affiliations:** School of Biological Sciences, University of Manchester, Manchester, United Kingdom

## Abstract

In 1961, the National Institutes of Health (NIH) began to circulate biological preprints in a forgotten experiment called the Information Exchange Groups (IEGs). This system eventually attracted over 3,600 participants and saw the production of over 2,500 different documents, but by 1967, it was effectively shut down following the refusal of journals to accept articles that had been circulated as preprints. This article charts the rise and fall of the IEGs and explores the parallels with the 1990s and the biomedical preprint movement of today.

## Introduction

Since 1991, physicists and mathematicians have been using the arXiv preprint repository to circulate articles and ideas, to the envy of many biologists. After a number of failed attempts, including ClinMed Netprints (1999–2005) and Nature Precedings (2007–2012), 2 biology preprint servers were launched in 2013—PeerJ Preprints and bioRxiv (Cold Spring Harbor Laboratory). Many journals will now consider an article that has appeared on a preprint server, and grant-awarding bodies on both sides of the Atlantic allow preprints to be cited in grant and fellowship applications—some, such as the Chan Zuckerberg Initiative, insist that their investigators deposit their papers as preprints. [1]

This is widely seen as an example of biology finally catching up with physics [2, 3]—it seems certain that the success of arXiv was influential in finally convincing journals to accept biology preprints. In fact, biology first adopted large-scale circulation of preprints over half a century ago, as part of a generalized interest in preprints that spanned much of science. From 1961–1967, the National Institutes of Health (NIH) in the United States pioneered a system known as the Information Exchange Groups (IEGs). The IEGs, forgotten except by a handful of historians of documentation [4,5,6,7], have been the subject of only 1 investigation, published as an unrefereed report in 1971 [8]. The IEGs have not been systematically studied by science historians—not only is there no IEG archive, there is not even a record of the documents they produced. The IEGs eventually fell victim to a campaign by journals and learned societies, who considered the organized circulation of preprints in both biology and physics to be a threat to their financial interests and to their perceived status as guardians of scientific integrity [9].

This article outlines the rise and fall of the IEGs and tells the cautionary tale of the ability of scientific publishers and entrenched interests within the academic community to restrict the sharing of knowledge.

## Launching the IEGs

**Fig 1:**
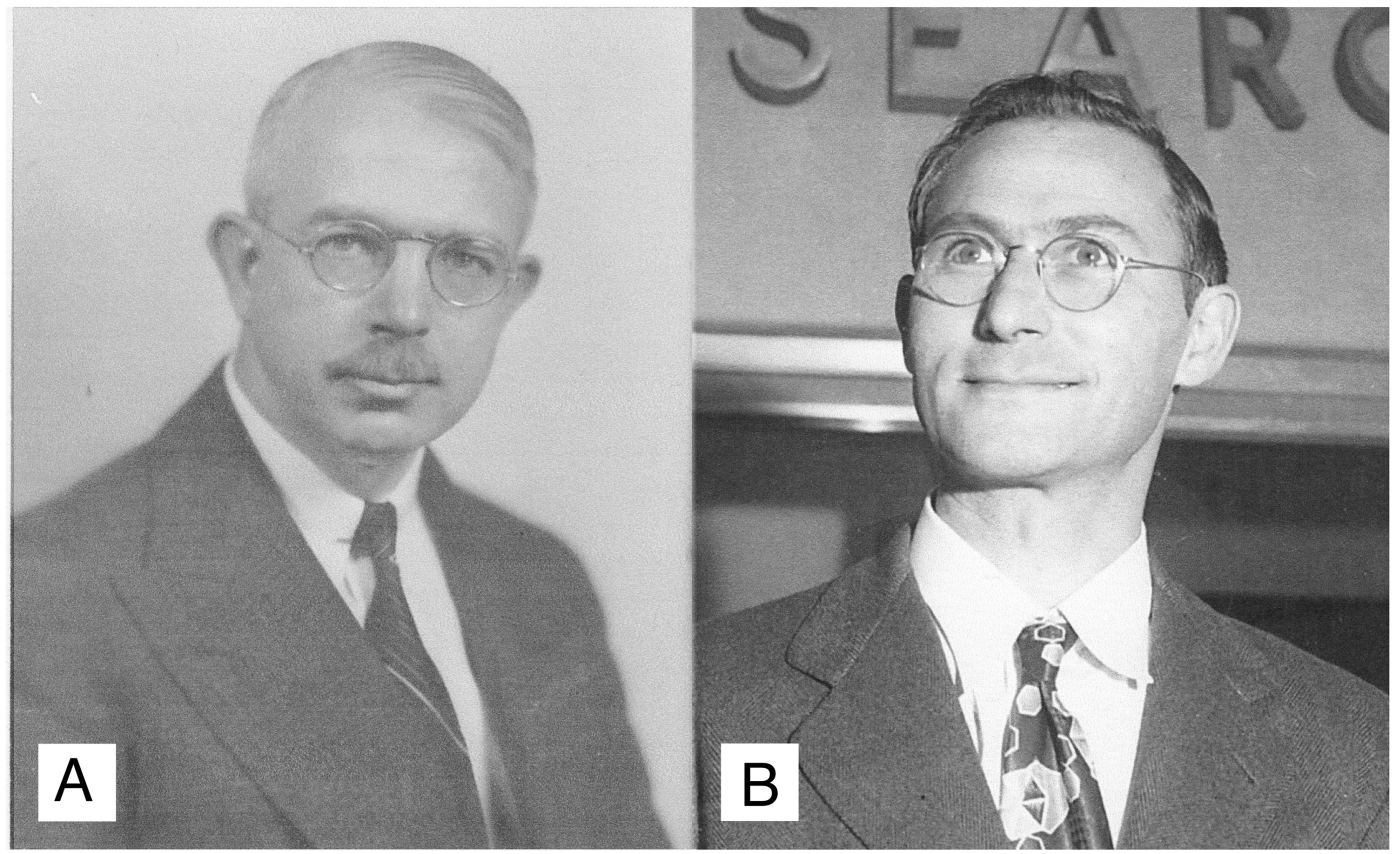
(A) Errett C. Albritton, MD (1890–1984), in 1948. Biographical information about Albritton is scant. For much of his career he was Professor of Physiology at the George Washington University Medical School, where he specialized in nutrition science. He later joined the NIH, where he worked in the Office of Research Accomplishments. In 1961, aged 70, he became the cofounder of preprints in the biosciences. Credit: Himmelfarb Health Sciences Library, George Washington University. (B) David E. Green, PhD (1910–1983), in 1961. Green was a biochemist at the University of Wisconsin–Madison, focusing on oxidative phosphorylation. This was the topic of the first IEG, which he cocreated and described as ‘one of the most revolutionary innovations in the history of science communication’ [9]. A biographical memoir described Green as ‘one of the giants of 20th century biochemistry…a complex person who had an extraordinary personality’. It makes no mention of his support for preprints [14]. Courtesy of the University of Wisconsin–Madison Archives (ID S14597). IEG, Information Exchange Group; NIH, National Institutes of Health.

**Fig 2:**
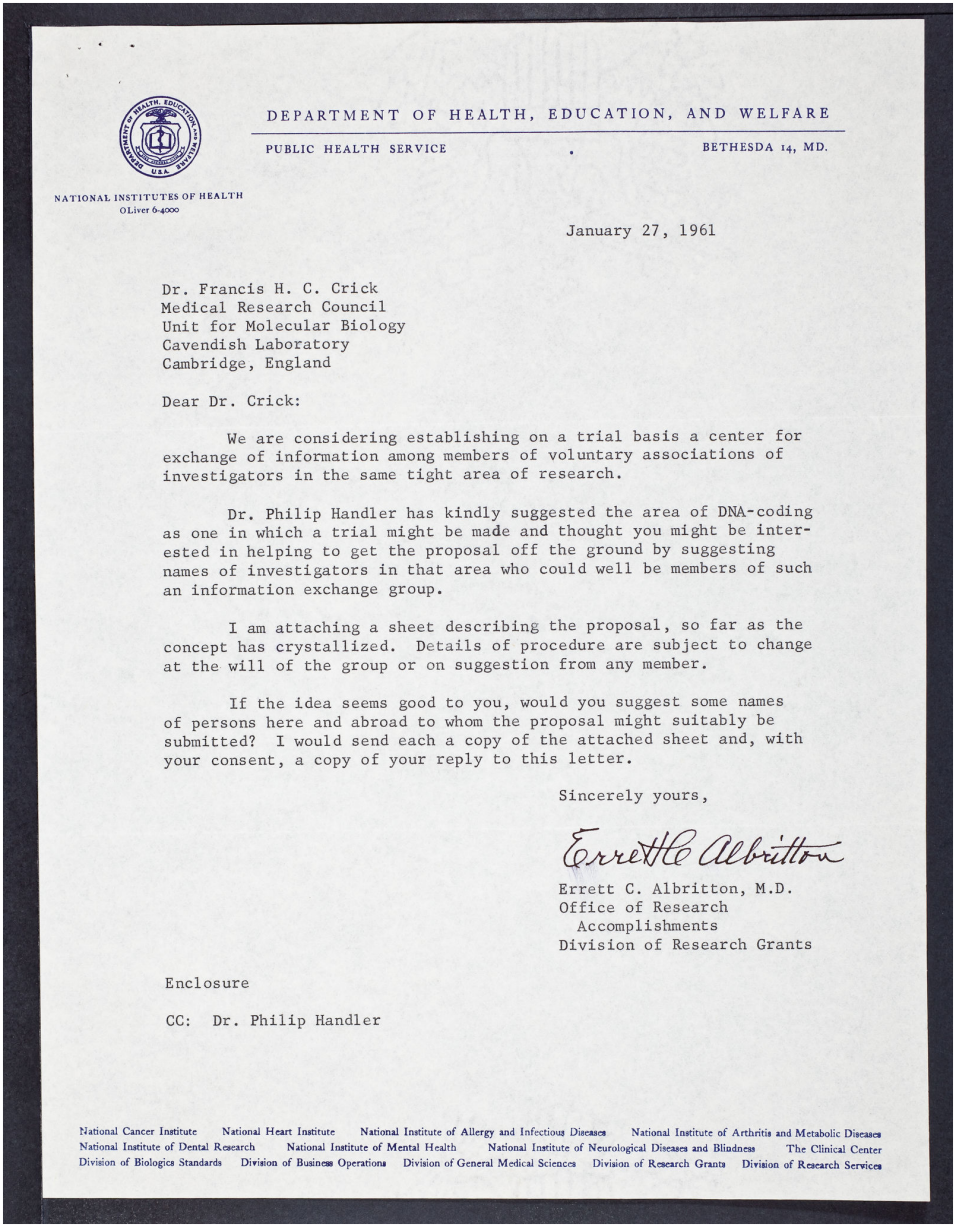
Letter from Albritton to Crick, January 1961 [10]. Credit: Cold Spring Harbor Laboratory Archive.

In 1961, Francis Crick received a letter from Errett C. Albritton, a 70-year-old administrator at the NIH (Figs 1A and 2), inviting him to join an informal network for circulating preprints called an IEG [10]. Crick gave Albritton the brush-off, saying he was ‘strongly opposed’ to the scheme [11], even though he had spent much of the previous 6 years circulating his own informal papers in such a network called the RNA Tie Club [12]. ‘There is far too much careless and rapid communication already in every area of this field of study’, Crick replied. ‘The idea of increasing it even in this semi-public manner fills me with horror’. Albritton’s response was good humoured (‘If it would not be a service to the area it needs a speedy burial!’ [13]), but Crick’s hostility was not widely shared, and there were enough positive responses for the first IEG to be set up shortly afterwards.

The IEG concept had been dreamt up in January 1961 by Albritton, along with 2 biochemists—David Green (Fig 1B) of the University of Wisconsin–Madison and Philip Handler of Duke University [15]. Albritton later described the IEGs as an ‘experiment’ or a ‘natural history study’ that would enable researchers working on a tightly focused research area to send ‘any communication whatever’ (preprint, comment, discussion, etc.) to the NIH, where the ‘memo’ would be physically reproduced and then circulated by the postal service to all members of the network. All costs were met by the NIH. Although the initial proposal was focused on a slightly cliquey group of ‘leading investigators’ [16], IEG membership was soon broadened to anyone ‘above the level of graduate student’, although the IEG chair had the final say on who could join and become a ‘subscriber’ [17]. Although memos were not supposed to be cited without permission, they could be taken as evidence of priority. The IEGs were intended to increase informal communication between scientists and to avoid the delays imposed by traditional publication methods. Albritton’s conception of the IEG was summarized by a brief slogan that was included on the front cover of each memo: it was a ‘continuing international congress by mail’ [15].

At one level, there was nothing new about circulating unrefereed documents or preprints. Previous systems were generally linked to specific institutions, such as the MIT Research Laboratory in Electronics that began producing unrefereed technical reports in 1946 [6] or the preprints circulated by the Petroleum Chemistry Division of the American Chemical Society from 1921 [18]. Other sets of unrefereed documents were tightly focused on the needs of a particular research community, such as the *Drosophila Information Service* [19], or were collected and sometimes distributed by institutional libraries, particularly in physics. Albritton’s NIH proposal was far more ambitious. It involved systematically circulating copies of all submitted preprints to a group of subscribers, rather than issuing them on request from an institution [20]. The scale of this experiment was immense, given the technology of the time: by the end of 1965, 3,663 researchers, from 46 different countries, were involved, and 2,561 different memos had been physically mailed out, involving millions of pages of paper [8].

The first IEG was focused on oxidative phosphorylation and terminal electron transport. It initially had only 32 members but grew to 386 within 4 years [8]. The IEG1 chair, David Green, underlined the advantages of the system: ‘The exchange makes it possible for all of its members to be fully informed in record time of all important developments in the field’ [21]. Other advantages included avoiding the danger of being ‘ambushed by some overzealous or overopinionated reviewer’, thereby providing ‘an outlet for anyone who feels choked by editorial intransigence’ [22]. Green insisted that, despite the lack of review, the IEG memos did not consist of a ‘flood of rubbish’; indeed, it was possible that informal review via the IEG might lead to a reduction in the number of weaker articles submitted to journals.

In October 1963, Albritton began soliciting suggestions for more IEGs and approached Sydney Brenner, Jacques Monod, and many others [23]. Like Crick 2 years earlier, Brenner gave a negative response: ‘the informal contacts that already exist facilitate enough exchange of information’, he wrote [24]. However, 5 new IEGs were soon created, covering Hemostasis (IEG2), Computer Simulation of Biological Systems (IEG3), Molecular Basis of Muscle Contraction (IEG4), Immunopathology (IEG5), and Interferon (IEG6). IEG7, focused on Nucleic Acids and the Genetic Code, was launched in early 1966 by Jim Watson and Marshall Nirenberg. Over 1,100 scientists immediately signed up [8]. Crick’s hostility to the IEG project diminished, and by October 1965, he was proposing Brenner and others as members of the future IEG7, although he warned Albritton that having multiple copies of IEG documents ‘pouring into our laboratory is more than we can stomach’ [25]. Among the most significant memos submitted to IEG7 was Francis Crick’s ‘wobble hypothesis’ explanation of codon–anticodon binding [26,27].

Overall, about 80% of the IEG memos were articles. Around one-third of these were circulated after acceptance by a journal but before publication; the remainder were submitted to the IEG before peer review and would be what we would now classify as preprints. There were also technical notes and—occasionally—debates. Over one-third of IEG members were from outside the US (mainly from the United Kingdom, Japan, and Australia), and over 90% of the memos were in English [8]. According to David Green, the system enabled researchers outside the US, including some in communist countries, to be as clued up about recent developments as their North American colleagues [9].

## The publishers strike back

The 1960s marked a period of substantial growth in scientific publishing, in particular through the activities of Pergamon Press, set up by the British businessman Robert Maxwell. The number of journal titles published by Pergamon rose from 40 in 1959 to 150 in 1965; while some were created as money-spinners, others were learned society journals that Pergamon took over [28]. The financial model that now dominates scientific publishing, with large numbers of for-profit journals paid for by institutional library subscriptions, began at this moment [29].

At this time, there were repeated discussions in the scientific community about the slowness of publication and the need for more informal and automated methods of communication [30], including a Ciba Foundation conference on the topic [31] and a report by the US President’s Scientific Advisory Committee [32]. Historians and librarians explored the consequences of the IEG for collaboration [18,33], while an influential article in the *Bulletin of the Atomic Scientists* argued that scientists in all fields should set up IEGs [34]. For several years, the library at the Stanford Linear Accelerator Center (SLAC) had collected preprints in high energy physics from around the world, as had the library at the European Organization for Nuclear Research (CERN). In 1965, the theoretical physicist Michael Moravcsik proposed formalizing these local initiatives, such that in each area of physics a central registry should collate all preprints and then regularly send out a list of the items that had been received (something like this was being operated at the Brookhaven National Laboratory) [35]. Within months, Charles Gottschalk of the US Atomic Energy Commission proposed the creation of a Physics Information Exchange (PIE). As Moravcsik explained in *Physics Today*, PIE was not completely analogous to the IEGs but was close enough that ‘some comfort can be gathered from the success IEG has encountered among biologists’ [36]. PIE would have a crucial cost-cutting difference—a single copy of each preprint would be sent to participating libraries, rather than to each individual member [36-37].

The growth of preprint circulation in all fields of science led some journal publishers—both commercial companies and learned societies—to feel that their prestige in the scientific community and their finances could be menaced. The counteroffensive began in April 1966 at a meeting of the American Association of Immunologists (AAI). Since 1916, the AAI had published *The Journal of Immunology*, and it clearly felt threatened by the creation of IEG5 (Immunopathology) [38], which had gained over 600 members and had produced over 300 memos in little more than a year [8]. The AAI meeting claimed that the circulation of IEG memos by the NIH was an ‘improper’ activity for a government agency, while the fact that memos were in reality ‘complete publications’ meant that they posed ‘a real danger’ to immunological journals and might ‘ultimately supersede them’. By a majority of 56 to 39, the AAI meeting voted that the publication of articles that had been previously circulated by IEG5 ‘should not be continued’ [38].

**Fig 3:**
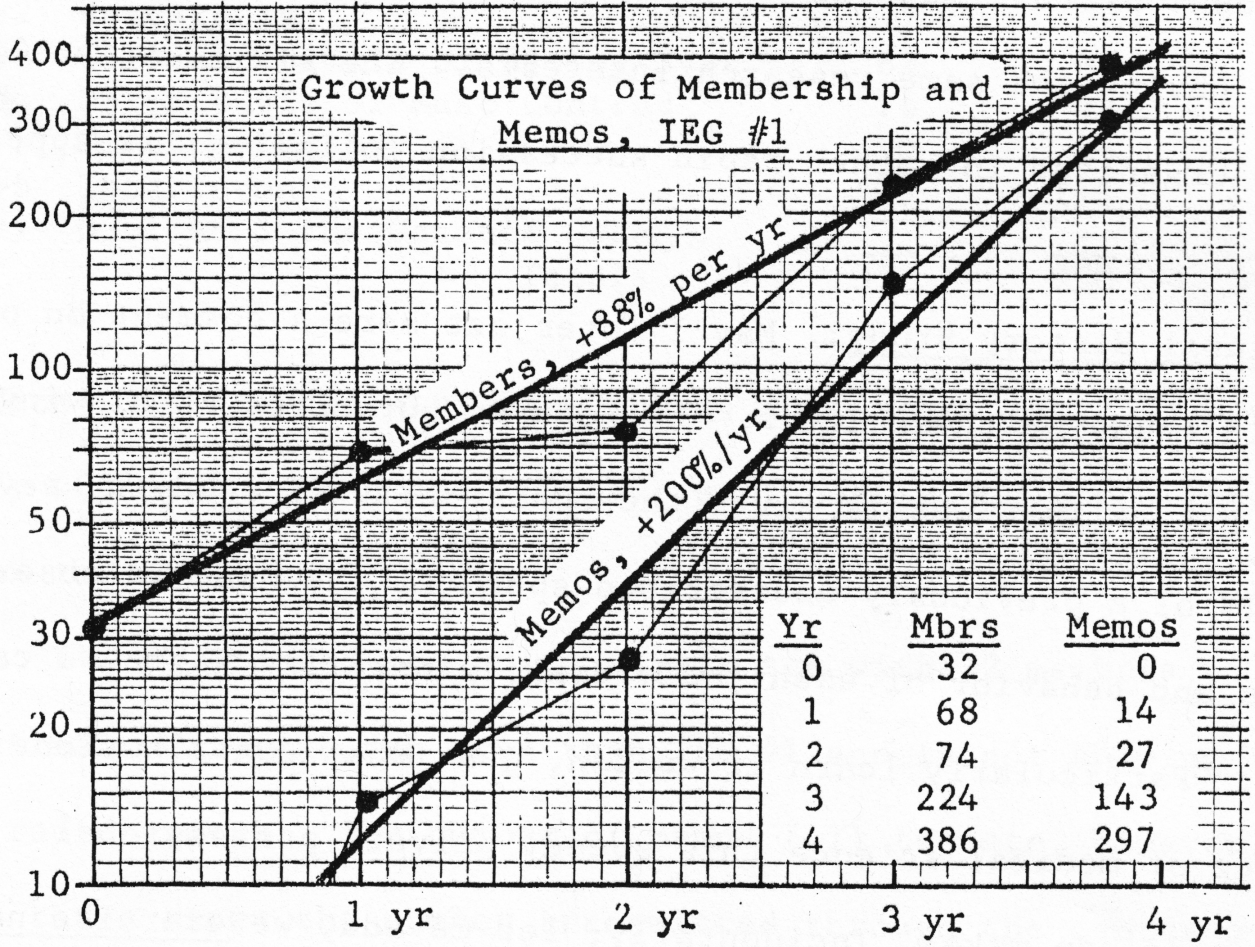
Growth of IEG1 1961–1965, as reported by Albritton [15]. Credit: Cold Spring Harbor Laboratory Archive. IEG, Information Exchange Group.

The massive growth in IEG membership (Fig 3) and the looming possibility of PIE, coupled with the hostility of the AAI to the IEGs, prompted *Nature* to wade into the debate. It was not that journal’s finest hour. In a series of articles and editorials in July and August 1966, including the unapproved reproduction of one of Albritton’s documents [39], *Nature* attacked the growth of the IEGs and the PIE proposal in sometimes sarcastic terms [40,41]. *Nature’s* first target was PIE—a proposal the journal considered to be ‘so offensive’ that it hoped it would be ‘stillborn’. The opening of one editorial, particularly condescending and alarmist, revealed the concern of the commercial publishers: ‘Next to downright villainy, misguided zeal is one of the most dangerous forces in society,’ they wrote [40].

Next in *Nature’s* sights were the IEGs, which a few weeks later were attacked by the journal as ‘suspect’ and a waste of money, as well as for being ‘in the publication business’ no matter what the NIH might claim. The defects of preprints, thundered the journal, included ‘inaccessibility, impermanence, illiteracy, uneven quality, and lack of considered judgment’ [41]. The traditional journal system had by contrast ‘encouraged thoroughness and measured judgment [and] discouraged triviality and repetitive work’.

This claim that journals act as guarantors of scientific quality was a key part of *Nature*’s criticism, as was the issue of priority. *Nature* was particularly irked by the fact that IEG members agreed to treat the memos as priority-laden. As Albritton put it: ‘a paper sent through the IEG is better protected than one published without prior circulation through the IEG’ [15]. Inevitably, financial considerations were also to the fore. A fraction of the money lavished on circulating preprints, argued *Nature*, should be devoted to ‘helping the journals become more efficient’. The for-profit journal was suggesting that the NIH should keep out of ‘the publication business’ and instead use that money to help commercial journals. The editorial closed with the same tone it had used throughout its coverage: ‘If the National Institutes of Health are as well-disposed towards the cause of effective publication as they seem to be, they could do a lot to help. The energy they choose to dissipate in Dr Allbritton’s print shop will be a lot less valuable’ [41].

A similarly aggressive attitude was adopted by the editor of *Science*. Philip H. Abelson suggested the products of the IEGs could be seen as ‘government-subsidised shoddy merchandise’ and concluded that, while there was an understandable frustration with ‘the inefficiency of many publications’, the IEGs also revealed ‘a desire on the part of some scientists to avoid a discipline essential to the integrity of science’ [42].

The fate of the IEGs was sealed not by the leading gatekeepers of scientific publishing but by a group of specialist journal editors. In September 1966, editors of leading biochemical journals met in Vienna to discuss the widespread circulation of preprints by the IEGs. There were 13 journals represented at the meeting, including the *Journal of Chemical Biology* and the *Journal of Molecular Biology* [43]. Like the AAI, this group decided—mostly without consulting their societies or editorial boards [8]—that no article that had been circulated as an IEG memo would be accepted for publication. It is striking that these journals and those published by the AAI overlapped with the 2 IEG areas that had the largest memberships: immunopathology and molecular biology, which together represented nearly 2,000 researchers.

This decision was soon leaked to *Nature*—an editorial crowed ‘Preprints made outlaws’ and praised the ‘firm…lethal steps’ the Vienna meeting of journal editors had taken against the IEG system [44]. The editorialist was right: no one would submit a preprint to an IEG under these conditions. Faced with the inevitable, the NIH caved in, and in November 1966, the head of the NIH Division of Research Grants, Eugene Confrey, announced that the IEGs would be closed the following March [45]. Albritton accepted that the IEGs were not financially viable without external funding [15], and growth in the number of preprints meant the IEGs were stretching the NIH’s financial and physical resources. Each copy of a memo cost $0.10–$0.50; by 1967, the IEGs were projected to cost the NIH $400,000 per annum, or over $3 million in today’s values [8,45].

Meanwhile, the letters pages of *Science* [46] and *Nature* [47,48] began to bulge with positions for and against IEGs. In *Science*, Philip Siekevitz, a cell biologist at Rockefeller University, claimed that the IEGs were ‘a dangerous nuisance’, while *Nature* pointed out in a note that although it had received 7 letters in support of the IEGs and only 1 against, Theodore Spaet, the Chair of IEG2, had encouraged its members to write in.

After the IEGs had been killed off, *Nature* produced a slightly more considered editorial entitled ‘Secret colleges end’ [49]. The journal recognized that there were problems of slowness and rigidity in the traditional journal format but insisted that, if successful, the IEGs ‘would have been an offence against scholarship’. The *New England Journal of Medicine* followed suit, going so far as to praise the ‘morally sensitive scientists’ who had opposed the IEGs before finishing on a contradictory note by calling for the IEG idea to be taken up again once the lessons had been learned [50]. The journal’s real position on preprints was made clear 2 years later, when it stated it would not accept any articles that had been previously published, including by ‘controlled-circulation journals’ [51]. Strict application of this principle, known as the Ingelfinger Rule after the journal’s editor and initially focused on preprints and media coverage, was subsequently extended to prevent the journal from publishing material that had appeared on any kind of website [52].

The PIE proposals met a similar fate. They were vigorously opposed by Simon Pasternack, the editor of *The Physical Review*, who described the project as ‘a great disservice’ [53]. Pasternack denied that PIE would be any quicker than traditional publication routes and predicted it would ‘dilute orderly communication and add confusion’. Going into rhetorical overdrive, Pasternack claimed PIE threatened physics research communication with ‘obscurity, incompleteness, polemics, inadequate references, discursiveness and irresponsibility’. Samuel Goudsmit, the editor of *Physical Review Letters*, joined in, producing a series of editorials in which he described a centralized register of preprints as ‘highly undesirable, as it would raise the unrefereed and unedited preprint to virtually the same status as a formal publication’ [54], emphasised the value provided by journals and peer review [55], and argued against citing preprints [56].

PIE was not stillborn as Pasternack and *Nature* wished, but it was instead launched for a trial year, functioning primarily as an announcement service of new preprints and discussion documents that was circulated to a mailing list; anyone interested had to request the document directly from the author.

## After the IEGs

After minor pushback [57] and some policy discussion of the significance of the experiment [31], most of the IEGs immediately folded. Albritton had hoped that because many IEG members—and even some IEG Chairs—were also Editors or Associate Editors of journals (including the *Journal of Molecular Biology* and the *Journal of Biological Chemistry*), peaceful coexistence with traditional journals would be possible [15]. This turned out to be naive. The power of the Vienna editors’ meeting and of the AAI, coupled with the hostility of *Science* and *Nature* and the financial strain on the NIH, stopped the IEGs in their tracks. Only IEG6 decided to keep going, after a 93% positive vote of its members. The 250-strong group continued to circulate material until at least the late 1970s under the title Interferon Scientific Memorandum. To reduce costs, their memos were restricted to 8 pages and distributed as reduced-size photos, with the support of the American Institute of Biological Sciences [58].

The perception of the IEGs by those who had been involved was overwhelmingly positive. Professor Michael Woodruff of the University of Edinburgh chided *Nature* for its ‘timid’ attitude; he found his membership of IEG5 to be of ‘enormous value’ and was ‘most delighted’ with reading and writing memos [59]. Surveys of IEG members showed 94% of the respondents said reading a memo had positively influenced a research decision, while 68% considered that the memos had saved time and money [8]. However, in most cases, the key memos were articles that eventually appeared in print; although the IEGs increased the rapidity and efficiency of communication, there was no evidence that it led to greater debate, one of the Albritton’s key objectives. In this respect, the IEGs failed.

Unbowed, Albritton’s colleagues at the NIH continued to emphasize the value of preprints [20]. In an understandably embittered article reviewing the rise and fall of the IEGs, David Green, the chair of IEG1 and cocreator of the scheme, decried the ‘strangulation’ of what he considered to be ‘one of the most revolutionary innovations in the history of science communication’ [9]. After dismissing the 3 criticisms leveled at the IEGs by the Vienna meeting and by *Science* and *Nature* (duplication, copyright infringement, and potential misunderstandings from lack of review), Green explained why the IEGs had really been killed off:

It is my opinion that the stated reasons are not the real reason. Rather, the stated reasons merely hide the fact that the editors were apprehensive that the status and prestige of the journals would be downgraded if another agency (IEG) were distributing to its members, from 6 months to a year earlier than the journals, the very papers which would eventually appear in the journals, though not necessarily in the same final form.

*Nature’s* final statement on the affair, made in February 1967, suggested that preprints should be renamed ‘impersonal communication’ or ‘postal circular’ and reiterated the ‘offense’ the IEGs had given to the established journals because of the claimed potential of duplicate publications. However, the editorialist was also keen to turn his article into an advert, reassuring his readers that the rapid circulation that was so attractive a feature of the IEGs would soon be found at *Nature*, which in a few months would ‘be operating consistently with a time lag of a few weeks’. The aim was for *Nature* to ‘beat the IEG at their own game’ [60].

Debate about how to enable more rapid communication of scientific discoveries in all fields continued into the 1970s [61,62]. The solution was finally found in physics, which already had established and successful local networks for collecting and distributing preprints. In January 1969, a new service, Preprints in Particles and Fields, was run out of SLAC. It built on the preprint services run by Lawrence Radiation Laboratory at Berkeley and the SLAC library and drew lessons from the fate of PIE and the IEGs [63]. Within a year, there were around 1,600 subscribers, showing the appetite for preprint circulation.

Over the next 2 decades, publishing was transformed as rapid progress in information technology enabled the development of increasingly rich and cost-effective schemes for circulating information. In 1991, Paul Ginsparg at the Los Alamos National Laboratory created an automated email server for distributing preprints, a system that eventually became known as arXiv [64]. In subsequent years, the impact of the World Wide Web, which was launched at virtually the same time as arXiv, transformed global communication and publishing, including the circulation of preprints. Initially set up for high energy physics, arXiv gradually extended into other fields and was soon partly supported by National Science Foundation (NSF) funding [3,4]. The concerns of a number of scientific societies and publishers were placated by the gradual growth of the system and the evident fact that it did not damage journal prestige or finances [5].

Life science researchers, who had either forgotten the IEG affair or never knew of it, could not help but notice the growth of arXiv. In 1999, Harold Varmus, in his final months as head of the NIH, became what he later described as a ‘radical proponent’ of new ways of circulating scientific information [65]. After informal discussions with leading biomedical scientists, Varmus proposed the creation of e-Biomed, an electronic repository of preprints that was clearly modelled on arXiv [52,65]. Varmus opened a consultation on his proposal and received overwhelming support from the individual scientists who responded to his call, but the journal publishers were deeply hostile and lobbied extensively against his scheme [65].

An editorial in the *New England Journal of Medicine* warned of ‘a potential threat to the evaluation and orderly dissemination of new clinical studies’—the journal was concerned that potentially incorrect clinical papers would gain the imprimatur of the NIH’s authority and could have significant negative consequences for patient health and well-being (this remains a worry for medical preprints) [66]. But the journal also revealed that one of its major concerns was the ‘probably disastrous effects’ on the paid circulation of journals. The Federation of American Societies of Experimental Biology (FASEB), a powerful umbrella group of learned societies, even threatened to use their lobbying power in Congress to affect the NIH budget should the e-Biomed proposal go ahead [52].

Within 4 months, the project was dead in the water. Varmus accepted that his vision could not be fully realized in the face of such opposition and focused instead on open access provision of accepted manuscripts through PubMed Central. This development, together with the legacy of the e-Biomed initiative, played a role in the development of the open access movement and the launching of PLOS by Varmus, Pat Brown, and Michael Eisen [65]. Nevertheless, it would be another decade and a half before biologists, their funders, and their editors accepted what had become commonplace in most parts of physics.

A third attempt in over 50 years to introduce preprints into biology occurred in 2013 with the launch of PeerJ Preprints and bioRxiv, following a series of initiatives by Ron Vale and others [1,2]. This time around, there appears to have been a shift in opinion amongst funders and publishers of biomedical research—there has not been the kind of hostility that appeared in the 1960s and 1990s.

This apparent change in attitude has yet to be systematically analysed, but here are some potential explanations, which are not mutually exclusive:

- The interval from submission to the first journal to final publication (perhaps in another journal) can be of the order of many months, similar to that in the 1960s. Coupled with the short duration of postdoctoral posts and the increasingly rapid development of technology, this has led to growing frustration with the ‘glacial pace’ of publication and a determination on the part of researchers to find a better solution [67].
- The new biological preprint servers have made clear that they are not encroaching on journal territory or finances but simply decoupling first dissemination of knowledge from the ‘certification’ that is represented by peer review.
- The widespread adoption of open access publishing and the free circulation of data and ideas may make opposing preprints simply look churlish in the age of the Internet and the success of arXiv.
- Finally, despite initiatives such as the San Francisco Declaration on Research Assessment [68], many key decisions affecting the lives of scientists—recruitment, promotion, and funding—continue to be based on the titles of the journals we publish in, rather than a direct estimation of the quality of the research we produce. In such a world, the journal will not go extinct—indeed, journals can make money by charging for open access, and can scout out promising papers on the preprint servers.

Whatever the case, on the third attempt, it appears that a culture of preprints has been established in the biosciences, although not yet in medicine. The fate of the IEGs should warn us of the power of commercial publishers and of vested academic interests to restrict the free circulation of knowledge.

The digital culture we now live in is far beyond the dreams of Errett C. Albritton and his printed and stapled IEG memos, individually sent out in the mail to eager subscribers. But he envisioned the importance of the open circulation of knowledge and debate over half a century ago. His name and his ambitions may have been forgotten, but he would recognize our world.

## Abbreviations

AAI: American Association of Immunologists
CERN: European Organization for Nuclear Research
FASEB: Federation of American Societies of Experimental Biology
IEGs: Information Exchange Groups
NIH: National Institutes of Health
NSF: National Science Foundation
PIE: Physics Information Exchange
SLAC: Stanford Linear Accelerator Center
